# Liver and Kidney Injuries in COVID-19 and Their Effects on Drug Therapy; a Letter to Editor

**Published:** 2020-03-09

**Authors:** Ali Rismanbaf, Sara Zarei

**Affiliations:** 1 Department of Clinical Pharmacy and Pharmacy Practice, School of Pharmacy and Pharmaceutical Sciences, Isfahan University of Medical Sciences, Isfahan, Iran.; 2 School of Medicine, Tehran Medical Sciences Branch, Islamic Azad University, Tehran, Iran.

**Keywords:** Coronavirus, Acute kidney injury, Liver Failure, Drug therapy

**Dear Editor,**

COVID-19 is a newly emerging human infectious disease of SARS-CoV-2 origin that has affected many countries around the world. COVID-19 is now rapidly spreading worldwide, and this letter is written as the World Health Organization (WHO) has declared a global emergency on January 31^st^ amid concerns about a growing outbreak of SARS-CoV-2. Most of the published articles on COVID-19 have highlighted lungs as the main organ involved in the disease, while few articles have reported SARS-CoV-2 involvement in other organs, including liver and kidneys, which can impair the metabolism and excretion of the medications taken to treat the disease.

According to Zhang et al. the incidence of hepatic abnormalities significantly increases after infection with COVID-19 and during the course of the disease, which may indicate the effect of SARS-CoV-2 on the liver or side effects of the medications used by patients (1). Also, Xu et al. have reported steatosis and liver injury in the liver biopsy of a patient with COVID-19 (2). In addition to liver injuries, some articles have also reported an increased incidence of acute renal injury following COVID-19, which could be due to the presence of SARS-CoV-2, the inflammation induced by the disease, or a synergistic effect of both on kidneys (3, 4). Additionally, Cheng et al. have reported that patients with acute renal injury have a higher mortality rate compared to other patients (3).

There is currently no definitive cure for COVID-19, and the treatment regimens prescribed for patients are the main treatments that have previously been effective in SARS-CoV and MERS-CoV, and chloroquine phosphate is the only medicine whose therapeutic effect has been proven by a clinical trial. Medicines currently prescribed to treat COVID-19 include Oseltamivir, Lopinavir / Ritonavir, Ribavirin, and Chloroquine Phosphate or Hydroxy Chloroquine Sulfate. All of these medicines are metabolized in the liver. Most of the metabolites derived from Oseltamivir, Ribavirin and some of the metabolites of Lopinavir / Ritonavir, Chloroquine Phosphate and Hydroxy Chloroquine Sulfate are found in the urine due to renal excretion. Therefore, injury to the liver and kidneys can impair metabolism, excretion, dosing and expected concentrations of the medications, which can increase the risk of toxicity using the aforementioned medications.

According to the reports, liver and kidneys can be damaged in patients with COVID-19, which may make reaching the therapeutic dose of the medicines difficult and increase the risk of adverse drug reactions in patients. As a result, frequent and careful monitoring of liver and kidney functions in patients with COVID-19 can lead to early diagnosis of liver and kidney disorders, and also help in achieving the optimal therapeutic concentrations and reducing the risk of adverse drug reactions.
